# Artificial intelligence-enabled electrocardiographic screening for left ventricular systolic dysfunction and mortality risk prediction

**DOI:** 10.3389/fcvm.2023.1070641

**Published:** 2023-03-03

**Authors:** Yu-Chang Huang, Yu-Chun Hsu, Zhi-Yong Liu, Ching-Heng Lin, Richard Tsai, Jung-Sheng Chen, Po-Cheng Chang, Hao-Tien Liu, Wen-Chen Lee, Hung-Ta Wo, Chung-Chuan Chou, Chun-Chieh Wang, Ming-Shien Wen, Chang-Fu Kuo

**Affiliations:** ^1^Division of Cardiology, Chang Gung Memorial Hospital, Taoyuan, Taiwan; ^2^Center for Artificial Intelligence in Medicine, Chang Gung Memorial Hospital, Taoyuan, Taiwan; ^3^School of Biomedical Informatics, University of Texas Health Science Center at Houston, Houston, TX, United States; ^4^School of Medicine, Chang Gung University, Taoyuan, Taiwan; ^5^Division of Rheumatology, Allergy and Immunology, Chang Gung Memorial Hospital, Taoyuan, Taiwan

**Keywords:** electrocardiogram, left ventricular systolic dysfunction, left ventricular ejection fraction, all-cause mortality, deep neural network

## Abstract

**Background:**

Left ventricular systolic dysfunction (LVSD) characterized by a reduced left ventricular ejection fraction (LVEF) is associated with adverse patient outcomes. We aimed to build a deep neural network (DNN)-based model using standard 12-lead electrocardiogram (ECG) to screen for LVSD and stratify patient prognosis.

**Methods:**

This retrospective chart review study was conducted using data from consecutive adults who underwent ECG examinations at Chang Gung Memorial Hospital in Taiwan between October 2007 and December 2019. DNN models were developed to recognize LVSD, defined as LVEF <40%, using original ECG signals or transformed images from 190,359 patients with paired ECG and echocardiogram within 14 days. The 190,359 patients were divided into a training set of 133,225 and a validation set of 57,134. The accuracy of recognizing LVSD and subsequent mortality predictions were tested using ECGs from 190,316 patients with paired data. Of these 190,316 patients, we further selected 49,564 patients with multiple echocardiographic data to predict LVSD incidence. We additionally used data from 1,194,982 patients who underwent ECG only to assess mortality prognostication. External validation was performed using data of 91,425 patients from Tri-Service General Hospital, Taiwan.

**Results:**

The mean age of patients in the testing dataset was 63.7 ± 16.3 years (46.3% women), and 8,216 patients (4.3%) had LVSD. The median follow-up period was 3.9 years (interquartile range 1.5–7.9 years). The area under the receiver-operating characteristic curve (AUROC), sensitivity, and specificity of the signal-based DNN (DNN-signal) to identify LVSD were 0.95, 0.91, and 0.86, respectively. DNN signal-predicted LVSD was associated with age- and sex-adjusted hazard ratios (HRs) of 2.57 (95% confidence interval [CI], 2.53–2.62) for all-cause mortality and 6.09 (5.83–6.37) for cardiovascular mortality. In patients with multiple echocardiograms, a positive DNN prediction in patients with preserved LVEF was associated with an adjusted HR (95% CI) of 8.33 (7.71 to 9.00) for incident LVSD. Signal- and image-based DNNs performed equally well in the primary and additional datasets.

**Conclusion:**

Using DNNs, ECG becomes a low-cost, clinically feasible tool to screen LVSD and facilitate accurate prognostication.

## Introduction

Heart failure (HF) is a major health issue affecting over 26 million people worldwide. It causes a significant increase in both morbidity and mortality and imposes a financial burden on society (1). Echouffo-Tcheugui et al. have classified left ventricular dysfunction into two categories: left ventricular systolic dysfunction (LVSD) and left ventricular diastolic dysfunction. LVSD is characterized by a reduced left ventricular ejection fraction (LVEF) and is associated with three times the risk of developing overt HF (2). Early identification of individuals with asymptomatic LVSD can lead to effective interventions, such as lifestyle changes, and medications, including angiotensin-converting enzyme inhibitors, angiotensin II receptor blockers, mineralocorticoid receptor antagonists, and beta-blockers (3–7), which can delay the onset of HF, reduce the rate of cardiac events, and improve survival (8–10).

The most commonly used method to assess LVSD is the transthoracic echocardiogram (TTE), but its limitations, including portability, cost, and operator dependency, restrict its use as a screening tool. To address this, there is a need for more accurate and accessible screening tools to identify LVSD in asymptomatic patients, such as a weighted scoring model incorporating clinical characteristics and plasma natriuretic peptides. However, these tools lack the specificity to predict LVSD in asymptomatic populations (11, 12).

The electrocardiogram (ECG) is an inexpensive and widely available method that measures the collective electrical activity of the heart and may contain information related to LVSD. While ECG recording is a standardized process, the accuracy and consistency of human interpretation can vary widely based on the experience and expertise of the interpreter. In addition, subtle ECG features that are invisible to the human eye may be useful for LVSD detection and prognostication. To overcome these challenges, the use of deep neural networks (DNNs) is proposed.

In recent years, DNNs have been applied successfully in the healthcare industry, including image analysis (13), predictive modeling (14), natural language processing (15), and drug discovery (16). They are superior to traditional pattern recognition methods (17) and form the foundation of clinical applications such as fracture detection (18), retinopathy grading (19), and lung nodule identification (20). DNN tools can interpret ECGs with similar accuracy to experienced physicians. Attia et al. developed a DNN-based ECG screening tool to identify individuals with LVEF ≤35% (21). A subsequent pragmatic clinical trial showed that a DNN-based intervention increased the likelihood of identifying patients with low LVEF during routine primary care (22). However, the effectiveness of DNN-based models in predicting incident LVSD and mortality has not been studied in a large clinical setting.

With data from approximately 1.7 million individuals, we conducted this study to evaluate the feasibility of using DNN-based ECG interpretation as a screening tool for LVSD and to assess its utility in risk assessment. The primary outcome was the ability of the DNN model to accurately identify individuals with LVSD (defined as LVEF <40%) based solely on the ECG. The secondary outcome was the ability of the DNN model to identify individuals at increased risk of death and at increased risk of developing LVSD.

## Materials and methods

### Data sources and study population

This study was conducted at Chang Gung Memorial Hospital (CGMH), the largest private hospital system in Taiwan. The study population included consecutive adult patients (age ≥ 18) who underwent standard 12-lead ECG at CGMH between October 2007 and December 2019 (1,777,039 individuals, 5,148,718 ECG tracings). ECGs with poor recording quality or unavailable leads were excluded. The ECG data were linked to the Chang Gung Research Database (CGRD), which included the electronic health records of all patients who visited any one of the following seven hospitals: Keelung, Taipei, Linkou (headquarters), Taoyuan, Yunlin, Chiayi, and Kaohsiung.

The patients’ survival status was confirmed by linking the CGRD to the National Death Registry. Valid internal patient record linkage was achieved by using unique patient identifiers, and these were encrypted before the data were released to researchers to protect patient confidentiality. This study was approved by the Institutional Review Board of CGMH and Tri-Service General Hospital. This study used anonymous and nontraceable data, so the need for patient consent was waived.

### Collection of data

Standard 12-lead ECGs with 10-s voltage-time traces were acquired at a sampling rate of 500 Hz using a MAC 5000, MAC 5500, or MAC5500HD ECG machine (GE Healthcare, Chicago, IL, United States) and stored using the Marquette Universal System for Electrocardiography (MUSE). Each standard 12-lead ECG was stored as a 12 × 5,000 matrix. Both the raw ECG signal data and processed ECG images at a 400 × 600-pixel resolution were obtained.

Transthoracic echocardiograms were performed and interpreted in accordance with the guidelines set forth by the American Society of Echocardiography and the American College of Cardiology/American Heart Association. Comprehensive two-dimensional (2D) or three-dimensional (3D) Doppler echocardiographic profiles and quantitative measurements were recorded in Chang Gung’s health information system. For this study, we only extracted LVEF values for analysis. LVEF was routinely measured using standardized methodologies. If different methods were used to measure LVEF in a report, the order of data preference was as follows: 3D echocardiogram, the Simpson biplane method, 2D method, linear measurement using M-mode. If multiple LVEF values were obtained using one method, the mean value was used for analysis.

To achieve proper correlation between ECG and TTE data, only TTEs obtained within 2 weeks of the index ECG were used for DNN model creation.

### Development of DNN models for identification of LVSD

In this study, we implemented two types of DNNs using the Pytorch framework and Python 3.6. All training was performed on an NVIDIA DGX-1 platform with 8 V100 GPUs and 32 GB of RAM per GPU. For the DNN that used signal inputs (DNN-signal), we used the deep residual network (ResNet) (23) modified to fit the signal input (Supplementary Figure 1). We used a wider kernel for the first convolution layer compared with the original ResNet framework as used for images. This architecture used skip connections, which allowed information to pass directly to the next layer to avoid the degradation caused by deeper neural networks. The network consisted of a convolution layer followed by eight residual blocks. Each residual block contained two convolution layers. The output of the last block was fed into hybrid pooling because combining max- and average-pooling methods improved the generalization ability while reducing dimensionality (24, 25). The output of hybrid pooling was subsequently sent to a fully connected layer to perform the final classification. The output of each convolutional layer was followed by batch normalization for distribution normalization and fed into a rectified linear activation unit (26). Cross-entropy loss with an Adam optimizer (27) was used in the model. Dropout was applied to reduce the overfitting by breakup co-adaptation on the training data (28).

For the DNN using the image inputs (DNN-image), we prepared a 400 × 600-pixel image similar to standard 12-lead ECG images (Supplementary Figure 2) using the signal data (12 × 5,000 matrix). The resolution was determined by a series of experiments using different image resolutions. The images were fed to ResNet-18 (23), and the output layer had two classes (Softmax function). The validation set was used to optimize the network architecture and network hyperparameters. The DNN-signal and DNN-image used the same training and validation sets for model building and were tested on the same testing set. A receiver operating characteristic (ROC) curve was plotted to assess the performance. The model with the highest area under the ROC curve (AUROC) was selected as the final model. We used the validation dataset ROC to select optimal threshold for the probability of LVSD by applying the Youden index (J) method.

We further assessed the network performance in different age, sex, and comorbidity strata. The odds ratio (OR), sensitivity, and specificity were calculated for each strata.

### Division of dataset

**Figure 1:**
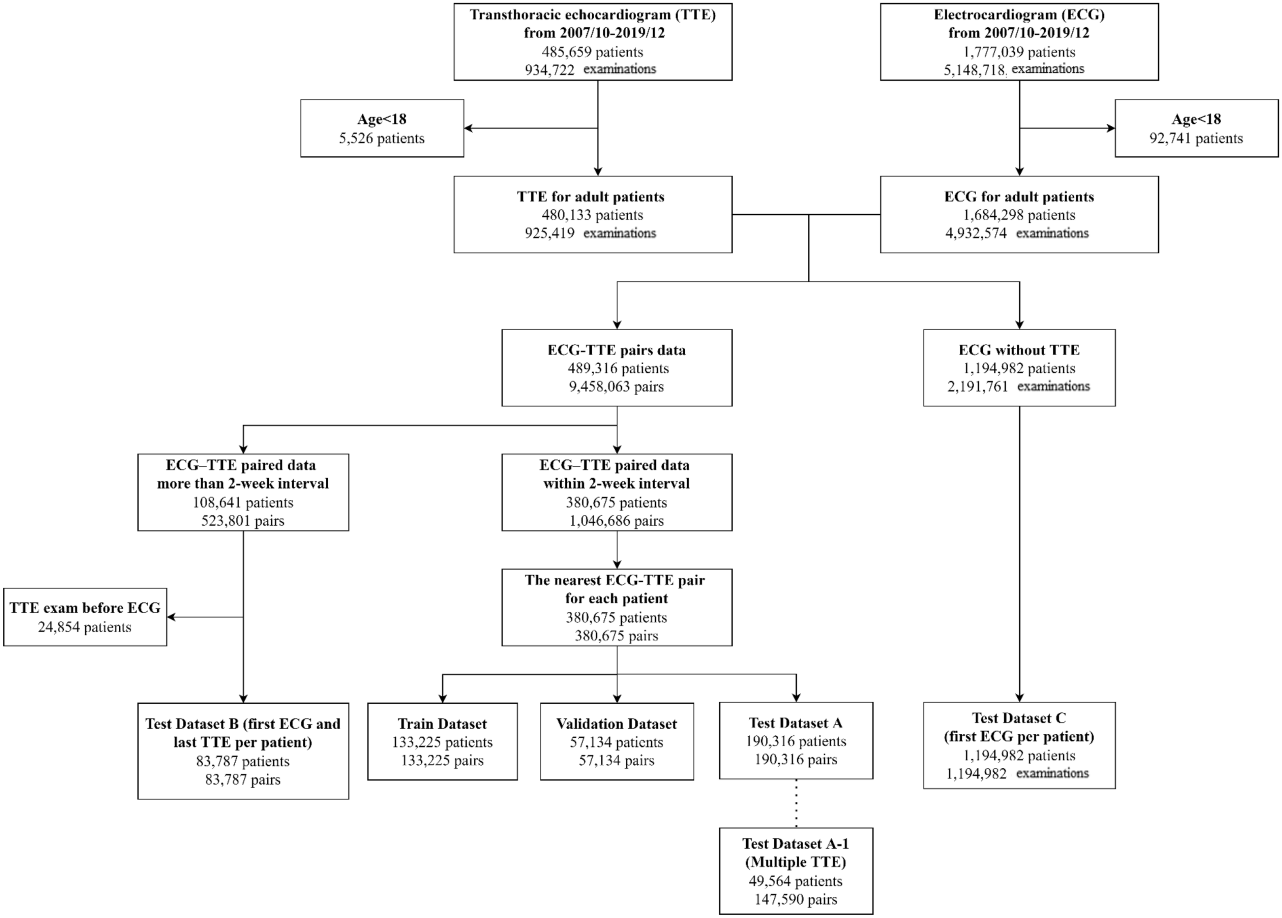
Data flow for ECG and TTE data pairing.

Among 1,684,298 adult patients with ECG tracings, 380,675 had at least one TTE data within 2 weeks of the index ECG during the study period (Figure 1). For patients with multiple ECG–TTE pairs, the earliest pair with the shortest ECG–TTE interval was selected for model development. Total 380,675 ECG–TTE paired datasets were used for the primary analysis. These ECG-TTE pairs were randomly allocated into a training, validation, or testing set using simple random sampling in which each dataset had an equal probability of selection without replacement. The final DNN development cohort included 133,225 patients in the training set, 57,134 in the validation set, and 190,316 in the testing set. No patient was allocated to more than one group (Figure 1).

We further conducted an external validation using paired ECG-TTE data from the Tri-service General Hospital. The external validation cohort included 91,425 consecutive adults between April 2010 and September 2021. The criteria of patient selection and echocardiographic performance methodology were the same as for the derivation cohort. Different from the ECG machine used at CGMH, ECGs from Tri-service General Hospital were obtained using the Philips system.

### Performance evaluation of the DNN models in predicting mortality

The ability of DNN to predict all-cause and cardiovascular mortality was assessed. According to the differences between the results of echocardiographic measurements and DNN predictions, we defined the following names: (i) ‘true positive’ DNN prediction represents both DNN-predicted and echo-measured LVEF <40%; (ii) ‘true negative’ DNN prediction represents both DNN-predicted and echo-measured LVEF ≥40%; (iii) ‘false positive’ DNN prediction represents DNN-predicted LVEF<40% and contemporaneous echo-measured LVEF ≥40%; and (iv) ‘false negative’ DNN prediction represents DNN-predicted LVEF≥40% and contemporaneous echo-measured LVEF <40%. The associations of different groups with all-cause or cardiovascular mortality were also assessed. The National Death Registry was linked to the study dataset. In Taiwan, it is mandatory for physicians to report deaths and causes of death to the Department of Health and Welfare. Therefore, death records within the National Death Registry are considered complete and accurate. A previous validation study estimated the effect of the misrecorded causes of death in the National Death Registry on cardiovascular mortality rates. The effect was less than 4%, suggesting accurate cause-of-death coding in Taiwan (29).

### Sensitivity analyses

We conducted sensitivity analyses in patients who were not included in the primary analysis. These patients were included in the following sub-analyses (Figure 1): (i) among patients with multiple TTE examinations in the original testing dataset (dataset A1, *n* = 49,564), the incidence of LVSD and mortality were compared in patients with ‘false-positive’ versus ‘true-negative’ predictions of LVSD; (ii) among patients who underwent TTE after more than 2 weeks of the index ECG (dataset B), the incidence of LVSD and mortality were compared in patients with positive versus negative predictions of LVSD; and (iii) among patients without echocardiographic data (dataset C), mortality rate was compared in patients with positive versus negative predictions of LVSD. Age- and sex-weighted Kaplan–Meier analysis was used to determine the incidence of LVSD or mortality. Cox proportional hazard regression was used to estimate the age- and sex-adjusted hazard ratios (HR; 95% confidence intervals [CI]) for LVSD and mortality.

### Statistical methods

Only the testing datasets were evaluated for performance measures. The model’s diagnostic performance was evaluated by calculating the AUROC, sensitivity, specificity, positive predictive value (PPV), and negative predictive value (NPV). The F1 score, harmonic mean of the PPV, and sensitivity based on the selected threshold were also computed. Continuous variables are expressed as means ± standard deviation (SD). Categorical variables are expressed as numbers and percentages. Adjusted odds ratios (OR; 95% CI) were calculated. For comparisons of population characteristics, the chi-square test was used for categorical variables and the unpaired Student’s t-test for continuous variables. Cox proportional hazards models were used to estimate hazard ratios (HR; 95%CI) for LVSD, all-cause, and cardiovascular mortality. A value of *p* < 0.05 was considered statistically significant. Statistical analyses were conducted using SAS 9.4 software.

## Results

The testing dataset contained 190,316 patients (46.3% females), and 8,216 patients (4.3%) had LVSD. The mean age was 63.7 ± 16.3 years. The median follow-up time was 3.9 years (interquartile range 1.5–7.9 years) for testing dataset. Table 1 shows the characteristics of the patients in the training, validation, and testing sets. There were no significant differences between groups.

**Table 1:** Patient characteristics and comorbidities.

Characteristics	Training (*n* = 133,225)	Validation (*n* = 57,134)	Testing (*n* = 190,316)	*P* value	Additional A-1 (*n* = 49,564)	Additional B (*n* = 83,787)	Additional C (*n* = 1,194,982)
Age years, mean ± SD	63.7 ± 16.3	63.8 ± 16.3	63.7 ± 16.3	0.480	65.9 ± 14.5	57.1 ± 15.8	50.6 ± 16.1
Age groups, n (%)				0.083			
<40	12,483 (9.4)	5,222 (9.1)	17,717 (9.3)		2,687 (5.4)	12,648 (15.1)	326,015 (27.3)
40–49	11,893 (8.9)	5,224 (9.1)	17,098 (9.0)		3,621 (7.3)	11,796 (14.1)	240,076 (20.1)
50–59	21,276 (16.0)	9,189 (16.1)	30,251 (15.9)		7,687 (15.5)	18,446 (22.0)	242,396 (20.3)
60–69	27,820 (20.9)	11,741 (20.5)	39,454 (20.7)		11,157 (22.5)	16,700 (19.9)	168,833 (14.1)
70–79	27,298 (20.5)	11,516 (20.2)	38,732 (20.4)		11,742 (23.7)	12,261 (14.6)	97,981 (8.2)
80+	32,455 (24.4)	14,242 (24.9)	47,064 (24.7)		12,670 (25.6)	11,936 (14.2)	119,681 (10.0)
Sex, n (%)				0.248			
Female	61,569 (46.2)	26,233 (45.9)	88,139 (46.3)		21,665 (43.7)	43,483 (51.9)	625,408 (52.3)
Male	71,656 (53.8)	30,901 (54.1)	102,177 (53.7)		27,899 (56.3)	40,304 (48.1)	569,574 (47.7)
EF, mean ± SD	66.8 ± 11.9	66.7 ± 12.0	66.8 ± 11.9	0.845	64.2 ± 13.9	69.4 ± 8.9	N/A
EF < 40%, n (%)	5,745 (4.3)	2,498 (4.4)	8,216 (4.3)	0.825	3,698 (7.5)	899 (1.1)	N/A
Medical history, n (%)							
Diabetes mellitus	37,454 (28.1)	15,898 (27.8)	53,608 (28.2)	0.277	18,730 (37.8)	18,034 (21.5)	127,042 (10.6)
Hyperlipidaemia	5,087 (3.8)	2,114 (3.7)	7,074 (3.7)	0.260	3,137 (6.3)	3,517 (4.2)	14,978 (1.3)
Renal disease	20,471 (15.4)	8,618 (15.1)	29,100 (15.3)	0.292	12,585 (25.4)	8,073 (9.6)	33,241 (2.8)
Hypertension	71,628 (53.8)	30,471 (53.3)	102,060 (53.6)	0.223	35,250 (71.1)	41,790 (49.9)	234,683 (19.6)
Coronary artery disease	28,741 (21.6)	12,309 (21.5)	41,077 (21.6)	0.980	20,394 (41.1)	12,018 (14.3)	20,569 (1.7)
Myocardial infarction	10,186 (7.6)	4,354 (7.6)	14,484 (7.6)	0.933	7,827 (15.8)	1,406 (1.7)	2,134 (0.2)
Without any	41,416 (31.1)	17,770 (31.1)	59,113 (31.1)	0.976	5,700 (11.5)	32,207 (38.4)	885,789 (74.1)
Death, n (%)							
Within 1 year	17,379 (13.0)	7,497 (13.1)	24,950 (13.1)	0.838	3,479 (7.0)	2,747 (3.3)	48,625 (4.1)
Within 3 years	27,541 (20.7)	11,786 (20.6)	39,264 (20.6)	0.954	8,868 (17.9)	6,459 (7.7)	80,156 (6.7)
Within 5 years	33,060 (24.8)	14,172 (24.8)	47,189 (24.8)	0.992	12,412 (25.0)	9,301 (11.1)	100,746 (8.4)
Anytime	39,440 (29.6)	16,917 (29.6)	56,290 (29.6)	0.981	16,727 (33.7)	15,297 (18.3)	139,905 (11.7)

*P* values were derived from ANOVA for comparisons of continuous variables and Pearson’s chi-squared test for comparisons of categorical variables.

### Performance of the DNN models in identifying LVSD

**Figure 2:**
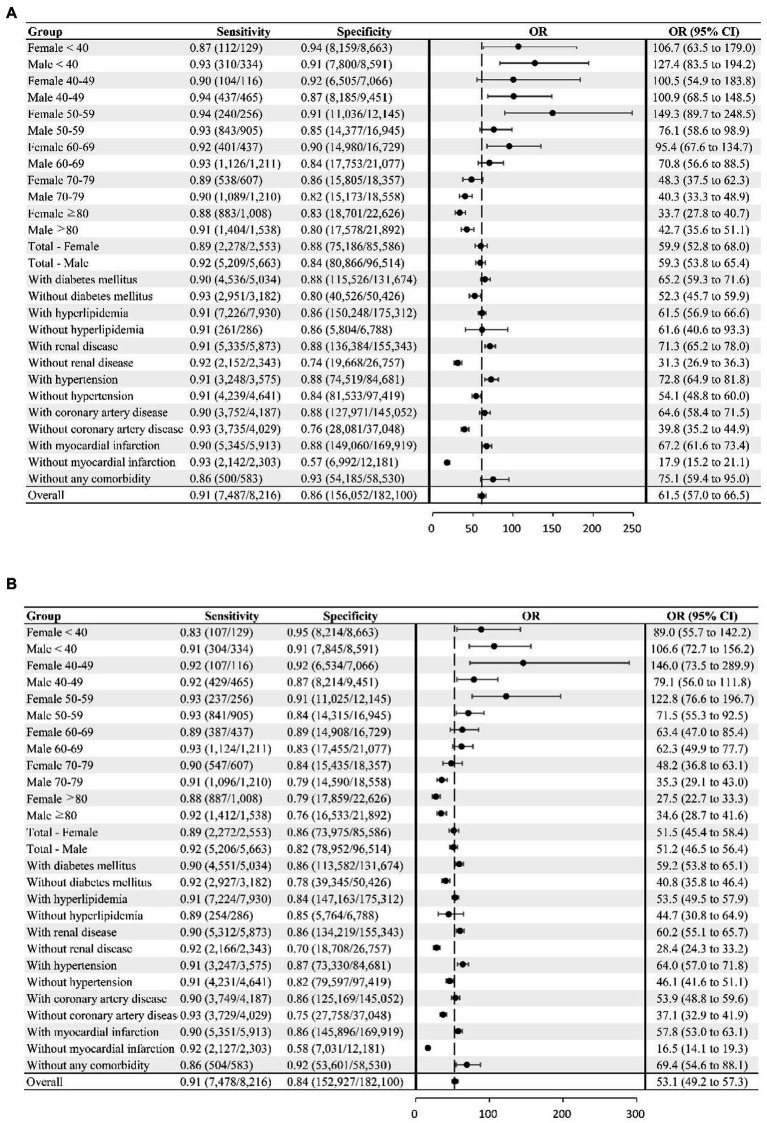
Deep neural network sensitivity, specificity, and odds ratio for detecting LVSD across different subgroups. The neural network’s sensitivity and specificity for detecting LVSD is tabulated across subgroups. The odds ratio (OR), which is the ratio of the positive ratio [sensitivity / (1−specificity)] to the negative likelihood [(1−sensitivity) / specificity], with the 95% CI, are shown for the subgroups and overall study sample. **(A)** LVSD prediction using signal. **(B)** LVSD prediction using image.

The AUROC values of DNN-signal and DNN-image for identifying LVSD in the testing dataset were 0.95 and 0.94, respectively (Supplementary Figure 3). When selecting a threshold maximizing the Youden’s index, the overall accuracy of DNN-signal was 0.86, with a sensitivity of 0.91, specificity of 0.86, PPV of 0.22 and NPV of 0.995. The DNN-image model performed with similar robustness to DNN-signal (sensitivity, 0.91; specificity, 0.84; PPV, 0.20; NPV, 0.995). The similarly robust DNN performances across different age, sex, and comorbidity strata in both DNN-signal and DNN-image are shown in Figure 2. External validation using ECG obtained by the Philips system was conducted. The AUROC of the DNN-signal for data from Tri-service General Hospital was 0.95. The overall accuracy of DNN-signal was 0.87, with a sensitivity of 0.90, specificity of 0.87, PPV of 0.19 and NPV of 0.99. Supplementary Tables 1, 2 show the patient characteristics and the performance of DNN-signal using data from Tri-service General Hospital.

### Performance of the DNN models in predicting mortality

**Figure 3:**
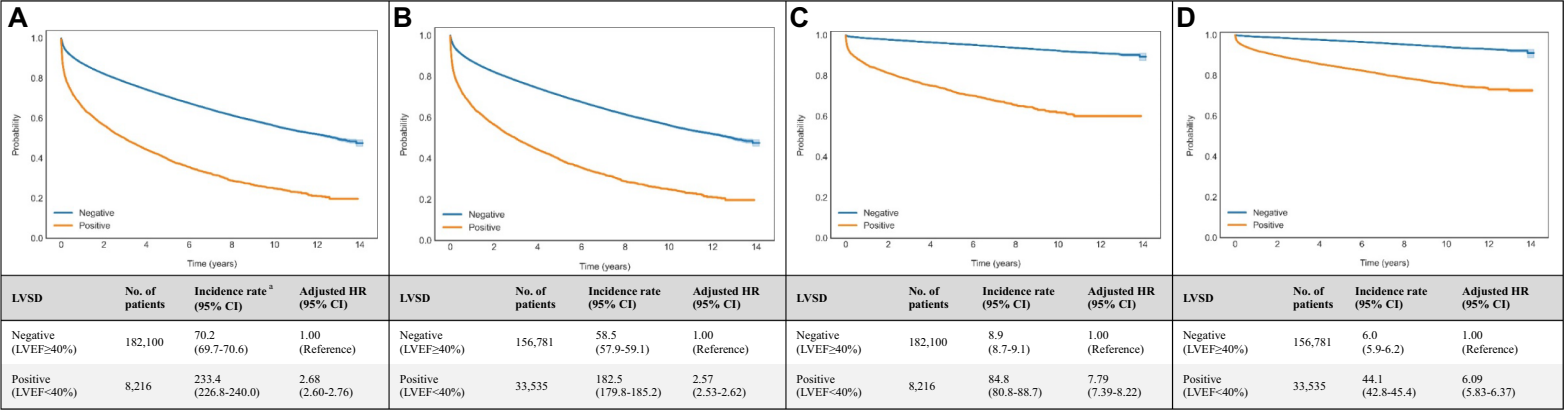
Associations of echocardiogram and DNN-signal predictions with all-cause and cardiovascular mortalities. Age- and sex-weighted Kaplan–Meier curves, death rates, and adjusted HRs (95% CI) stratified by **(A)** echo-derived LVSD for all-cause mortality (blue line, LVEF≥40%; yellow line, LVEF<40%), **(B)** DNN signal-predicted LVSD for all-cause mortality (blue line, LVEF≥40%; yellow line, LVEF<40%), **(C)** echo-derived LVSD for cardiovascular mortality (blue line, LVEF≥40%; yellow line, LVEF<40%), **(D)** DNN signal-predicted LVSD for cardiovascular mortality (blue line, LVEF≥40%; yellow line, LVEF<40%). ^a^ Adjusted K-M curves were adjusted by the inverse probability of treatment weighting, which calculated using sex and age. ^b^ The unit of incidence rate was 1,000 person-years. CI, confidence interval; DNN, deep neural network; LVEF, left ventricular ejection fraction.

Age- and sex-weighted Kaplan–Meier curves for mortality of patients with DNN signal-predicted LVSD and echo-derived LVSD are shown in Figure 3. A total of 8,216 LVSD patients were identified using echocardiographic data, and 33,535 LVSD patients were identified using DNN-signal. DNN signal-predicted LVSD was associated with age- and sex-adjusted HRs (95% CI) of 2.57 (2.53–2.62) for all-cause mortality and 6.09 (5.83–6.37) for cardiovascular mortality at a median follow-up of 3.9 years. Echo-derived LVSD was associated with age- and sex-adjusted HRs (95% CI) of 2.68 (2.60–2.76) for all-cause mortality and 7.79 (7.39–8.22) for cardiovascular mortality. The DNN-image performed similarly to DNN-signal with age- and sex-adjusted HRs (95% CI) of 2.70 (2.66–2.75) for all-cause mortality and 6.47 (6.19–6.77) for cardiovascular mortality (Supplementary Figure 4).

**Figure 4:**
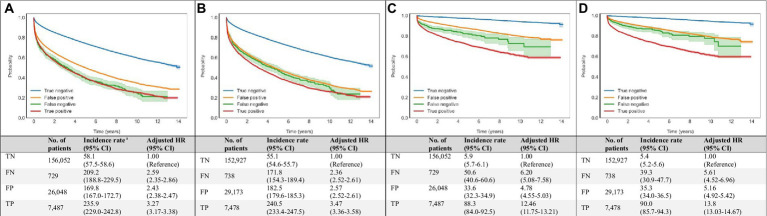
Associations of DNN-signal and DNN-image predictions with all-cause and cardiovascular mortalities. Age- and sex-weighted Kaplan–Meier curves, death rates, and adjusted HRs (95% CI) stratified by both echocardiography and DNN (true negative: blue line, both echo-measured and DNN-predicted LVEF ≥40%; false negative: green line, echo-measured LVEF <40% and DNN-predicted LVEF ≥40%; true positive: red line, both echo-measured and DNN-predicted LVEF <40%; and false positive: yellow line, echo-measured LVEF ≥40% and DNN-predicted LVEF <40%) for all-cause and cardiovascular mortality **(A)** DNN-signal predictions and all-cause mortality, **(B)** DNN-image predictions and all-cause mortality, **(C)** DNN-signal predictions and cardiovascular mortality, and **(D)** DNN-image predictions and cardiovascular mortality. ^a^ Adjusted K-M curves were adjusted by the inverse probability of treatment weighting, which calculated using sex and age. ^b^ The unit of incidence rate was 1,000 person-years. CI, confidence interval; DNN, deep neural network; EF, ejection fraction; FN, false negative; FP, false positive; HR, hazard ratio; K-M, Kaplan–Meier; LVSD, left ventricular systolic dysfunction; No., number; TN, true negative; TP, true positive.

Compared with ‘true negative’ DNN predictions, ‘true positive’ DNN-signal predictions were associated with HRs (95% CI) of 3.27 (3.17–3.38) for all-cause mortality and 12.46 (11.75–13.21) for cardiovascular mortality. ‘True positive’ DNN-image predictions were associated with HRs (95% CI) of 3.47 (3.36–3.58) for all-cause mortality and 13.8 (13.03–14.67) for cardiovascular mortality (Figure 4).

Among patients with ‘false positive’ DNN prediction, a higher mortality rate was also observed during follow-up. ‘False positive’ DNN-signal predictions were associated with HRs (95% CI) of 2.43 (2.38–2.47) for all-cause mortality and 4.78 (3.55–5.03) for cardiovascular mortality. ‘False positive’ DNN-image predictions were associated with HRs (95% CI) of 2.57 (2.52–2.61) for all-cause mortality and 5.16 (4.92–5.42) for cardiovascular mortality (Figure 4).

### Sensitivity analyses

Table 2 summarizes the performance of the DNN models in additional datasets. Subset A1 included 49,564 patients with multiple echocardiograms. Within this subset, ‘false positive’ DNN-signal predictions were associated with HRs (95% CI) of 8.33 (7.71–9.00) for incident LVSD, 1.99 (1.92–2.06) for all-cause mortality, and 3.51 (3.25–3.80) for cardiovascular mortality compared to ‘true negative’ DNN-signal predictions. ‘False positive’ DNN-image predictions were associated with HRs (95% CI) of 8.19 (7.57–8.87) for incident LVSD, 2.05 (1.98–2.12) for all-cause mortality, and 3.77 (3.49–4.07) for cardiovascular mortality compared to ‘true negative’ DNN-image predictions.

**Table 2:** Sensitivity analyses of model performance to identify patients with future left ventricular systolic dysfunction (LVSD) and those at risk of all-cause and cardiovascular mortalities.

Datasets/predictions	Incident LVSD		All-cause mortality		Cardiovascular mortality	
	Rate (95% CI)	HR (95% CI)	Rate (95% CI)	HR (95% CI)	Rate (95% CI)	HR (95% CI)
Subset A1: 45,866 patients with preserved LVEF by TTE in the testing dataset with multiple echocardiograms						
Negative by DNN-signal (*n* = 36,920)	8.6 (8.1–9.1)	1.00 (Reference)	54.6 (105.7–112.3)	1.00 (Reference)	7.9 (7.5–8.3)	1.00 (Reference)
Positive by DNN-signal (*n* = 8,946)	75.9 (72.3–79.5)	8.33 (7.71–9.00)	109.0 (105.7–112.3)	1.99 (1.92–2.06)	27.7 (26.1–29.4)	3.51 (3.25–3.80)
Negative by DNN-image (*n* = 35,604)	8.0 (7.5–8.5)	1.00 (Reference)	51.9 (50.9–52.9)	1.00 (Reference)	7.1 (6.7–7.5)	1.00 (Reference)
Positive by DNN-image (*n* = 10,262)	69.9 (66.7–73.2)	8.19 (7.57–8.87)	114.4 (111.3–117.6)	2.05 (1.98–2.12)	28.7 (27.1–30.3)	3.77 (3.49–4.07)
Subset B: 83,787 patients who had TTE > 14 days after index ECGs						
Negative by DNN-signal (*n* = 74,928)	1.5 (1.3–1.7)	1.00 (Reference)	22.9 (22.5–23.3)	1.00 (Reference)	1.9 (1.8–2.0)	1.00 (Reference)
Positive by DNN-signal (*n* = 8,859)	30.6 (28.2–32.9)	19.23 (16.56–22.33)	62.6 (60.5–64.8)	2.18 (2.09–2.26)	12.1 (11.2–13.1)	5.20 (4.70–5.75)
Negative by DNN-image (*n* = 73,795)	1.4 (1.2–1.5)	1.00 (Reference)	21.7 (21.3–22.1)	1.00 (Reference)	1.8 (1.6–1.9)	1.00 (Reference)
Positive by DNN-image (*n* = 9,992)	28.3 (26.2–30.5)	19.52 (16.72–22.80)	69.8 (67.7–72.0)	2.32 (2.24–2.41)	12.2 (11.3–13.1)	4.99 (4.52–5.52)
Subset C: 1,194,982 patients without TTE						
Negative by DNN-signal (*n* = 1,155,523)	–	–	16.9 (16.8–17.0)	1.00 (Reference)	1.1 (1.0–1.1)	1.00 (Reference)
Positive by DNN-signal (*n* = 39,459)	–	–	100.3 (98.8–101.8)	3.24 (3.19–3.29)	14.3 (13.7–14.9)	6.83 (6.51–7.16)
Negative by DNN-image (*n* = 1,151,691)	–	–	16.3 (16.2–16.4)	1.00 (Reference)	1.0 (1.0–1.0)	1.00 (Reference)
Positive by DNN-image (*n* = 43,291)	–	–	120.5 (118.9–122.2)	3.46 (3.40–3.51)	15.9 (15.4–16.5)	6.82 (6.51–7.14)

Data are rates (95% CI) and HRs (95% CI) of LVSD, all-cause mortality, and cardiovascular mortality. CI, confidence interval; DNN, deep neural network; ECG, electrocardiogram; HR, hazard ratio; K-M, Kaplan–Meier; LVEF, left ventricular ejection fraction; LVSD, left ventricular systolic dysfunction; TTE, transthoracic echocardiogram.

Within subset B, including 83,787 patients, positive DNN-signal predictions were associated HRs (95% CI) of 19.23 (16.56–22.33) for incident LVSD, 2.18 (2.09–2.26) for all-cause mortality, and 5.20 (4.70–5.75) for cardiovascular mortality. Positive DNN-image predictions were associated HRs (95% CI) of 19.52 (16.72–22.80) for incident LVSD, 2.32 (2.24–2.41) for all-cause mortality, and 4.99 (4.52–5.52) for cardiovascular mortality.

Within subset C, including 1,194,982 patients, DNN signal-predicted LVSD was associated with a HR (95% CI) of 3.24 (3.19–3.29) for all-cause mortality and 6.83 (6.51–7.16) for cardiovascular mortality. DNN image-predicted LVSD was associated with a HR (95% CI) of 3.46 (3.40–3.51) for all-cause mortality and 6.82 (6.51–7.14) for cardiovascular mortality. Supplementary Figures 5–12 show Kaplan–Meier curves for incident LVSD, all-cause and cardiovascular mortality for subsets A1, B, and C.

## Discussion

The prevalence of LVSD ranges from 2 to 8% in adults depending on the study population and cut-off value used (8–10). In both symptomatic and asymptomatic cases, LVSD is associated with increased morbidity and mortality. The Framingham cohort study showed that individuals with asymptomatic LVSD (LVEF <40%) have around eight-fold increased risk of developing HF (30). The combination of definite treatment and primary prevention of incident HF can reduce the disease burden. One such strategy is to screen for asymptomatic LVSD; however, the best method for this is unclear (11, 31, 32). Our study demonstrated the potential of DNNs for screening asymptomatic LVSD. In addition, comprehensive real-world testing demonstrated the robustness of DNN to identify LVSD and patients at risk of future LVSD and mortality. Furthermore, we constructed DNN models based on both raw ECG signals and transformed images. In clinical settings in which raw ECG signals are not available, this method can digest ECG image tracing and provide similar performance. Consequently, the applicability of DNN-enabled ECG is broadened.

ECG is a ubiquitous and economical point-of-care diagnostic tool in cardiology. Previous research has demonstrated that LVSD might be characterized by specific ECG changes, such as Q-waves (33, 34), left bundle branch block (35), and wide QRS duration (>120 ms) (36). However, no single feature had high enough predictive value to offer clinical utility. These various features seemed to interact in a non-linear fashion that could not be accounted for by traditional statistical methods or algorithmic approaches. DNNs afford the ability to consider complex datasets in the context of all of the contained data rather than preselected discrete data elements. Identifying these features may offer novel findings that can provide new diagnostic approaches or therapeutic targets. Finding ways to understand what drives the network’s interpretation is also the direction of future efforts.

We used DNN algorithms to perform binary classification of LVEF in a hospital-based population, with excellent performance (AUROC, 0.95) superior to known screening tests (e.g., natriuretic peptides) (11). The DNN performed well across all age, sex, and comorbidity groups (Figure 2). In addition, the model performance was validated externally using data from the Phillips system, suggesting its robustness across different machine types. The diagnostic performance was characterized by a high NPV, which helps exclude LVSD with high confidence. The ‘false positive’ rates were high. However, we further demonstrated that ‘false positive’ DNN predictions were associated with an eight-fold increased risk of incident LVSD (confirmed by TTE), a two-fold increased risk of all-cause mortality, and a five-fold increased risk of cardiovascular mortality compared to ‘true negative’ DNN predictions. This means that DNN could detect early, subclinical, electrical or structural abnormalities shown on the ECG. These abnormalities may include cardiac arrhythmias, left ventricular deformation, valvular heart disease, or metabolic derangements and thus increase the risk of LVSD incidence and death. In this case, DNN-enabled ECG is an effective screening tool to identify patients at risk.

Several studies have demonstrated the potential of AI in turning ECGs into functional screening and diagnostic tools for various heart disorders. For instance, Mayo Clinic researchers have applied AI to automatically detect LVSD and even tried to identify atrial fibrillation through sinus rhythm. Compared with prior studies (21, 37), we not only verified the diagnostic effectiveness of AI-assisted ECG reading on LVSD screening, but also explored the use of ECGs as an outcome prediction tool with the assistance of AI. Individuals with a positive DNN prediction were associated with a two-fold increased risk of all-cause mortality and a six-fold increased risk of cardiovascular mortality at a median follow-up of 3.9 years. This finding suggested that some trivial electrical abnormalities due to metabolic or myocardial disturbances may precede LVSD. It was speculated that some of these disturbances might be irreversible or progressive, eventually causing long-term adverse effects.

While this study reveals that DNN-enabled ECG interpretation is a reliable method of detecting LVSD, the selection of target populations for screening remains to be addressed. Galasko et al. evaluated a variety of LVSD screening strategies and demonstrated that LVSD screening is more cost-effective in high-risk subjects than in the general population (38). High-risk subjects were defined as those with hypertension, diabetes, atherosclerotic cardiovascular disease, and heavy alcohol consumpton (39). Our research included individuals who visited the hospital for various reasons, not just for known heart disease. This hospital-based population did have higher prevalences of diabetes mellitus (28.2%), hypertension (53.6%), and coronary heart disease (7.6%), which fits the definition of a high-risk group.

Based on this study, we propose a prototype approach for in-hospital LVSD screening. Step one involves ECG screening using the DNN-enabled classification of individuals who will undergo high-risk invasive treatment or those with pre-existing cardiovascular risk. Step two involves TTE evaluation of individuals identified as abnormal by DNN models. This DNN-enabled screening strategy offers an advantage, as ECG machines and internet services are widely available in modern hospitals, and the strategy is also financially sustainable. This DNN model also provides a potential complementary care approach to plasma natriuretic peptide measurement for primary LVSD screening. Further studies are needed to assess the impacts of the proposed DNN-enabled screening strategy on the incidence and prognosis of in-hospital HF-associated adverse events. Furthermore, a comprehensive analysis may be conducted to examine the cost-effectiveness of the proposed strategy.

In summary, DNN-enabled ECG is a valuable tool to screen for LVSD and predict outcomes. Given the low cost of DNN-enabled ECG, serial screening is possible, which also helps optimize screening strategy for LVSD without using invasive laboratory testing, particularly in settings with limited medical resources.

### Limitations of the study

There are several limitations to this study. First, some of the LVEF data used for analysis were measured using M-mode way. The major limitation of M-mode is its one dimensional nature and lack of direct spatial information. When regional LV deformation exists, the M-mode-derived LVEF is not reliable. Although most operators choose the 2D or 3D methods when performing LVEF measurements in patients with structural heart disease, we cannot completely rule out this potential bias. Second, echocardiographic parameters other than LVEF, such as left ventricular diameter, left ventricular diastolic function, right ventricular function or valvular heart disease, also affect mortality risk. However, the present study did not introduce these parameters to analyze and evaluate their impact on prognosis. Further research should be conducted to assess the differences between clinical characteristics of patients with DNN-predicted LVSD compared to those without DNN-predicted LVSD. Third, the study was conducted in an academic medical center in patients with more complex diseases. The primary analysis consisted of patients with a higher prevalence of HF and other cardiovascular comorbidities, whom clinicians identified as needing a TTE evaluation. Considering these cohort characteristics, the findings may not be generalizable to relatively healthy and truly asymptomatic populations. To verify the generalizability of our DNN models, we conducted multiple additional analyses in more than 1 million patients with different clinical characteristics. In addition, the stratified analysis of patients without known comorbidities showed a similar performance of the models. Finally, although the sensitivity and specificity were both satisfying in our study, we observed a relatively lower PPV. The performance of PPV is highly correlated to the proportion of positive subjects in the testing group. The low likelihood of LVSD (4.3%) in testing dataset caused a low PPV. Despite this, an appropriate sensitivity is more critical in applying ECG as an LVSD screening tool. The purpose of this screening tool is to detect all potential subjects who are at risk of developing LVSD for following echocardiogram exams.

## Conclusion

The established DNN algorithms in this study enable rapid LVSD detection and represent an essential step in transforming the ECG into an effective, real-time screening tool. Its ability to predict LVSD incidence and long-term mortality may help stratify patient risk and initiate relevant interventions. With good accuracy and accessibility, DNN-enabled ECG has the potential to optimize the screening process for LVSD among at-risk populations and to advance HF care significantly.

## Data availability statement

The original contributions presented in the study are included in the article/Supplementary material, further inquiries can be directed to the corresponding author.

## Ethics statement

The studies involving human participants were reviewed and approved by the Chang Gung Medical Foundation—Institutional Review Board. Written informed consent for participation was not required for this study in accordance with the national legislation and the institutional requirements.

## Author contributions

M-SW and C-FK conceived and designed the study. Y-CHu and Y-CHs did the literature search, acquired data, and wrote the manuscript. C-HL, RT, and J-SC did the statistical analyses. Y-CHs and Z-YL developed, trained, and applied the deep neural network. J-SC prepared the figures and tables. C-FK accessed and verified the data. H-TL, W-CL, H-TW, P-CC, C-CC, C-CW, and M-SW provided the commentary. All authors contributed to the interpretation of data and the revision of the manuscript, and approved the final manuscript.

## Funding

This work was supported by the Ministry of Science and Technology of Taiwan (grant number MOST 109-2321-B-182A-007, MOST 110-2314-B-182A-123, and MOST 110-2745-B-075A-001) and Chang Gung Memorial Hospital (grant number CLRPG3H0013, CORPG3L0161, and CORPG3L0461). We were also given methodological assistance from the University of Nottingham.

## Conflict of interest

The authors declare that the research was conducted in the absence of any commercial or financial relationships that could be construed as a potential conflict of interest.

## Publisher’s note

All claims expressed in this article are solely those of the authors and do not necessarily represent those of their affiliated organizations, or those of the publisher, the editors and the reviewers. Any product that may be evaluated in this article, or claim that may be made by its manufacturer, is not guaranteed or endorsed by the publisher.

## Abbreviations

AI: artificial intelligence
DNN: deep neural network
ECG: electrocardiogram
HF: heart failure
LVEF: left ventricular ejection fraction
LVSD: left ventricular systolic dysfunction
TTE: transthoracic echocardiogram.
