# Supplementary material for: Artificial intelligence-enabled electrocardiographic screening for left ventricular systolic dysfunction and mortality risk prediction
Source: Front Cardiovasc Med. 2023 Mar 3;10:1070641. doi: 10.3389/fcvm.2023.1070641 (PMC10029758; doi:10.3389/fcvm.2023.1070641)
Supplement: Supplementary file 1 [file Table_1.docx]

**Supplementary materials**

**Supplementary Figure 1.** Schematic representation of the DNN model.


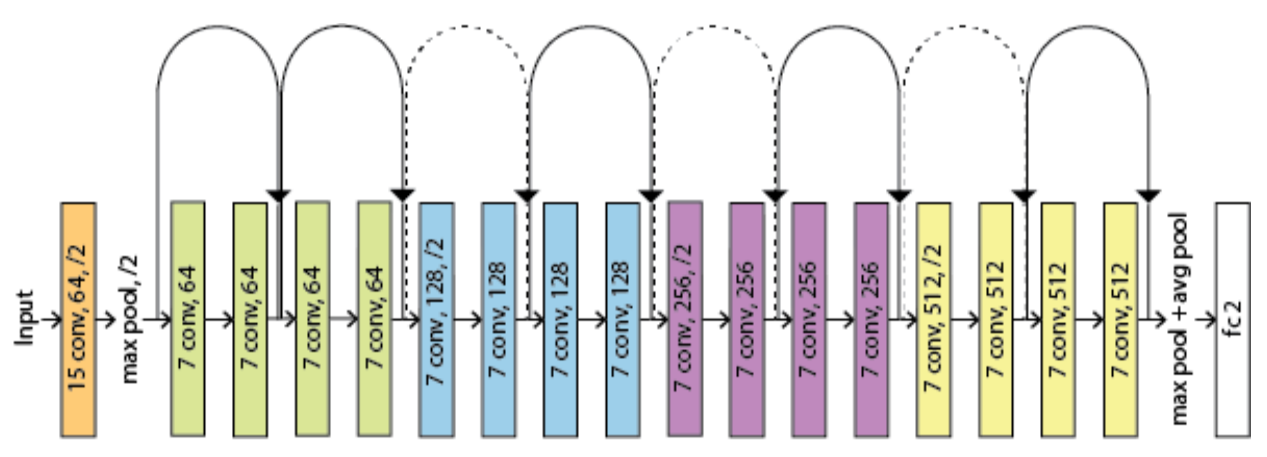


Abbreviation: DNN, deep neural network

**Supplementary Figure 2.** Sample electrocardiograms (400 × 600 pixels, all leads for 2.5 s and a long lead II for 10 s) from the raw signal data (12 × 5000 matrix).


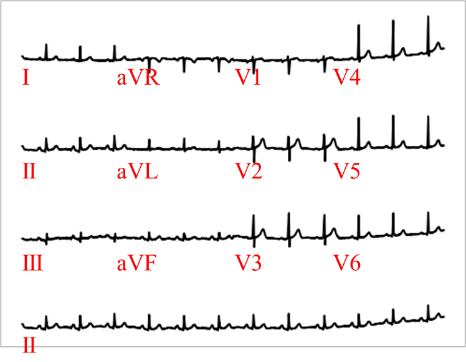


**Supplementary Figure 3.** Receiver operating characteristic curves and confusion matrices to identify left ventricular systolic dysfunction (LVSD) for the (a) DNN-signal model and (b) DNN-image model.

(a) DNN-signal model


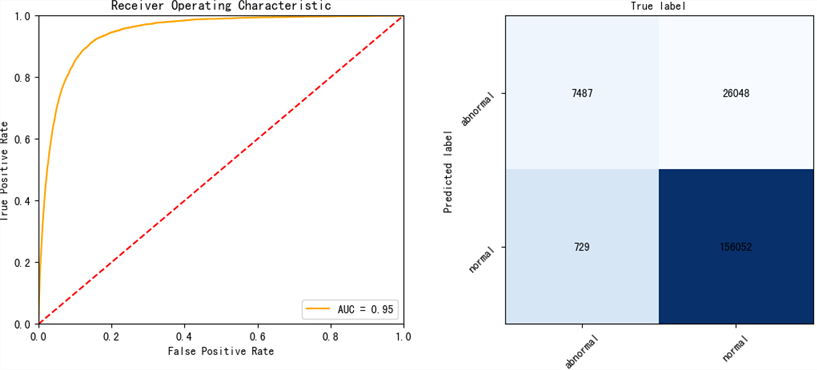


(b) DNN-image model


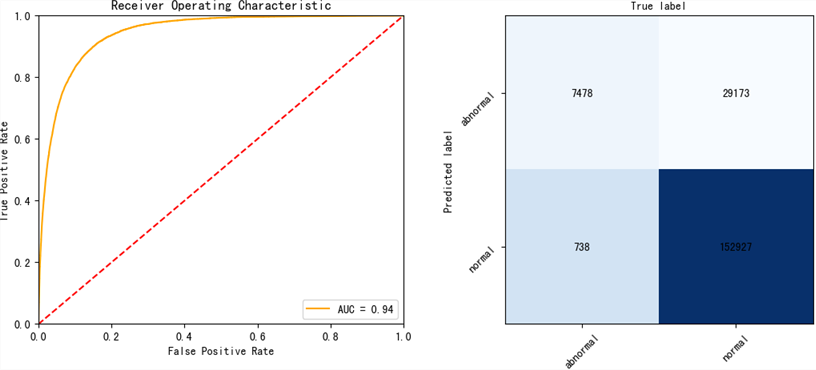

Abbreviations: AUC, area under curve; LVSD, left ventricular systolic dysfunction; ROC, receiver operating characteristics curve

**Supplementary Figure 4.** Associations of DNN-image predictions with all-cause and cardiovascular mortalities.

Age- and sex-weighted Kaplan–Meier curves, mortality rates, and adjusted HRs (95% CI) stratified by DNN-image, for (a) all-cause mortality (blue line, LVEF≥40%; yellow line, LVEF<40%), and (b) cardiovascular mortality (blue line, LVEF≥40%; yellow line, LVEF<40%).

| (a) | | | | (b) | | | |
| --- | --- | --- | --- | --- | --- | --- | --- |
| 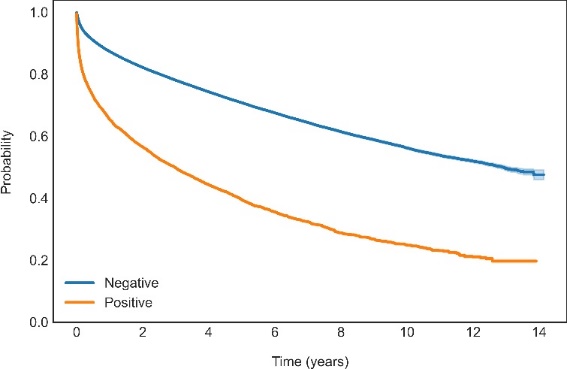 | | | | 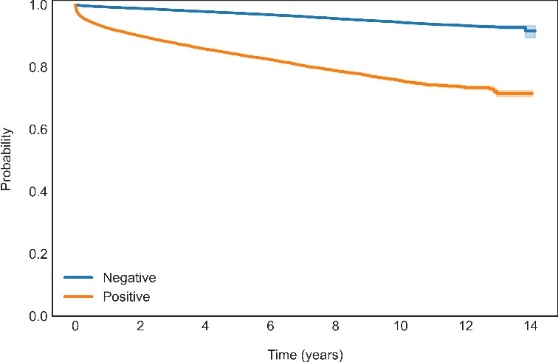 | | | |
|  | **No. of patients** | **Incidence rate** ^a^  **(95% CI)** | **Adjusted HR**  **(95% CI)** |  | **No. of patients** | **Incidence rate** ^a^  **(95% CI)** | **Adjusted HR**  **(95% CI)** |
| Negative (LVEF≥40%) | 153,665 | 55.5  (54.9-56.1) | 1.00  (Reference) | Negative (LVEF≥40%) | 153,665 | 5.5  (5.3-5.7) | 1.00  (Reference) |
| Positive (LVEF<40%) | 36,651 | 192.7  (190.1-195.4) | 2.70  (2.66-2.75) | Positive (LVEF<40%) | 36,651 | 45.0  (43.7-46.2) | 6.47  (6.19-6.77) |

^a^ The unit of incidence rate was 1,000 person-years.

Abbreviations: CI, confidence interval; DNN, deep neural network; HR, hazard ratio; LVEF, left ventricular ejection fraction; LVSD, left ventricular systolic dysfunction; No., number

**Supplementary Figure 5.** Age- and sex-weighted Kaplan–Meier curves for incident left ventricular systolic dysfunction (LVSD) among patients in subset A1 with preserved left ventricular ejection fraction (LVEF) stratified by (a) DNN-signal predictions and (b) DNN-image predictions.

| (a) Using signal | | | | (b) Using image | | | |
| --- | --- | --- | --- | --- | --- | --- | --- |
| 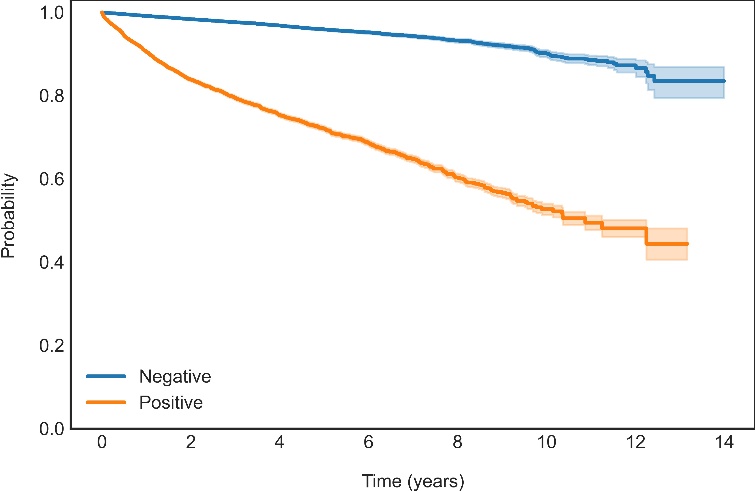 | | | | 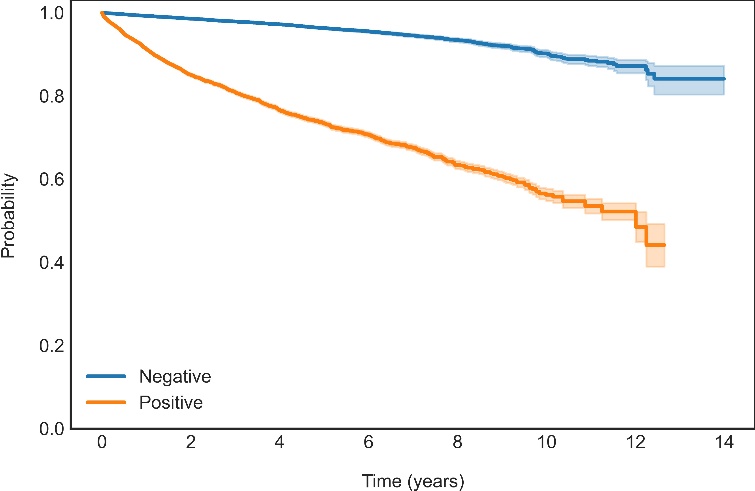 | | | |
|  | **No. of patients** | **Incidence rate** ^a^ **(95% CI)** | **Adjusted HR**  **(95% CI)** |  | **No. of patients** | **Incidence rate** ^a^ **(95% CI)** | **Adjusted HR**  **(95% CI)** |
| EF≥40% | 36,920 | 8.6 (8.1-9.1) | 1.00 (Reference) | EF≥40% | 35,604 | 8.0 (7.5-8.5) | 1.00 (Reference) |
| EF<40% | 8,946 | 75.9 (72.3-79.5) | 8.33 (7.71-9.00) | EF<40% | 10,262 | 69.9 (66.7-73.2) | 8.19 (7.57-8.87) |
| For the false-positive group, the number (%) of LVSD cases was 729 (8.2%) within 1 year, 1,281 (14.3%) within 3 years, and 1,483 (16.6%) within 5 years. For the true-negative group, the number (%) of LVSD cases was 289 (0.8%) within 1 year, 619 (1.7%) within 3 years, and 836 (2.3%) within 5 years. | | | | For the false-positive group, the number (%) of LVSD cases was 777 (7.6%) within 1 year, 1,368 (13.3%) within 3 years, and 1,600 (15.6%) within 5 years. For the true-negative group, the number (%) of LVSD cases was 241 (0.7%) within 1 year, 532 (1.5%) within 3 years, and 719 (2.0%) within 5 years. | | | |

^a^ The unit of incidence rate was 1,000 person-years.

Abbreviations: CI, confidence interval; DNN, deep neural network; FP, false positive; HR, hazard ratio; K-M, Kaplan-Meier; LVEF, left ventricular ejection fraction; LVSD, left ventricular systolic dysfunction; No., number; TN, true negative

**Supplementary Figure 6.** Age- and sex-weighted Kaplan–Meier curves for all-cause mortality among patients in subset A1 with preserved left ventricular ejection fraction (LVEF) stratified by (a) DNN-signal predictions and (b) DNN-image predictions.

| (a) Using signal | | | | (b) Using image | | | |
| --- | --- | --- | --- | --- | --- | --- | --- |
| 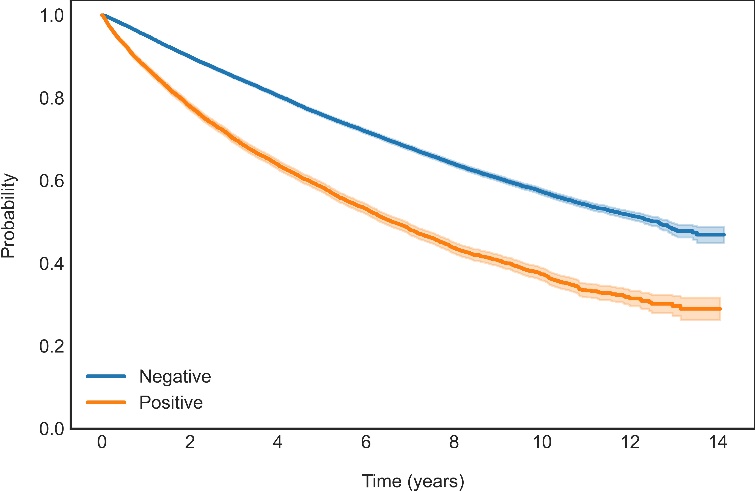 | | | | 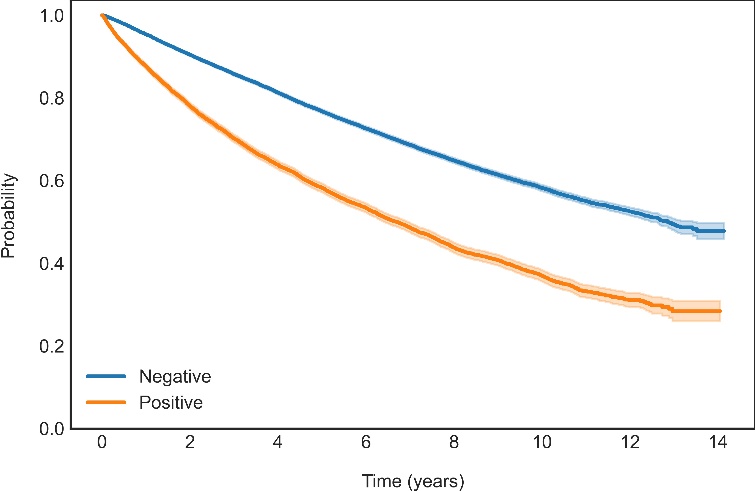 | | | |
|  | **No. of patients** | **Incidence rate** ^a^  **(95% CI)** | **Adjusted HR**  **(95% CI)** |  | **No. of patients** | **Incidence rate** ^a^  **(95% CI)** | **Adjusted HR**  **(95% CI)** |
| Negative prediction | 36,920 | 54.6 (53.6-55.6) | 1.00 (Reference) | Negative prediction | 35,604 | 51.9 (50.9-52.9) | 1.00 (Reference) |
| Positive prediction | 8,946 | 109.0 (105.7-112.3) | 1.99 (1.92-2.07) | Positive prediction | 10,262 | 114.4 (111.3-117.6) | 2.05 (1.98-2.12) |

^a^ The unit of incidence rate was 1,000 person-years.

Abbreviations: CI, confidence interval; DNN, deep neural network; HR, hazard ratio; No., number

**Supplementary Figure 7.** Age- and sex-weighted Kaplan–Meier curves for cardiovascular mortality among patients in subset A1 with preserved left ventricular ejection fraction (LVEF) stratified by (a) DNN-signal predictions and (b) DNN-image predictions.

| (a) Using signal | | | | (b) Using image | | | |
| --- | --- | --- | --- | --- | --- | --- | --- |
| 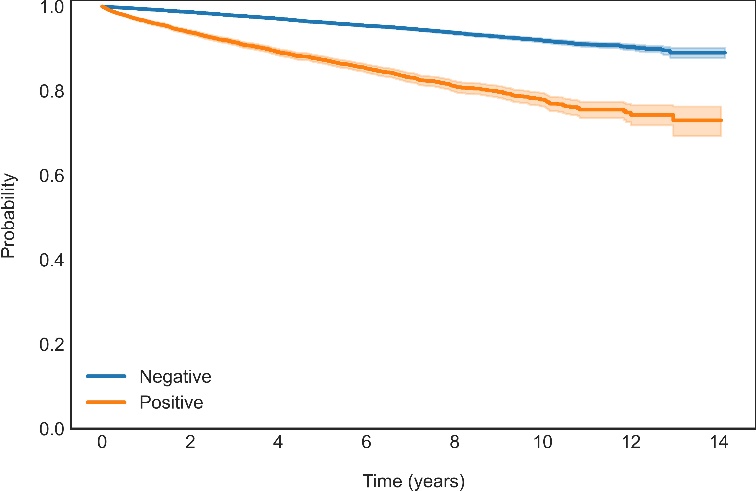 | | | | 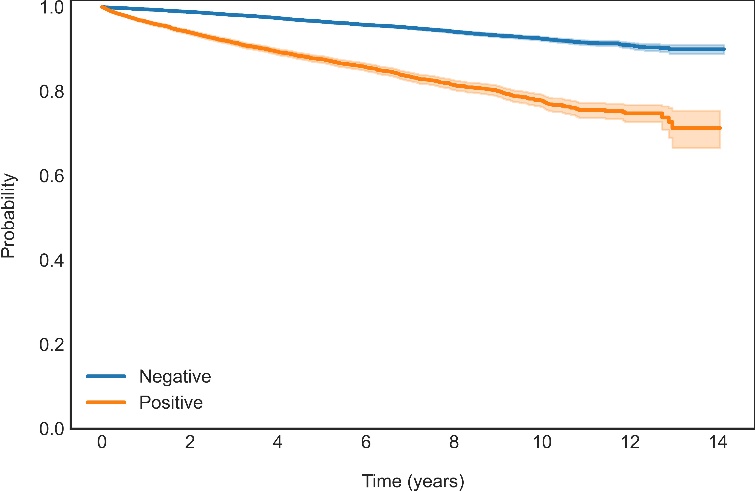 | | | |
|  | **No. of patients** | **Incidence rate** ^a^  **(95% CI)** | **Adjusted HR**  **(95% CI)** |  | **No. of patients** | **Incidence rate** ^a^  **(95% CI)** | **Adjusted HR**  **(95% CI)** |
| Negative prediction | 36,920 | 7.9 (7.5-8.3) | 1.00 (Reference) | Negative prediction | 35,604 | 7.1 (6.7-7.5) | 1.00 (Reference) |
| Positive prediction | 8,946 | 27.7 (26.1-29.4) | 3.54 (3.27-3.83) | Positive prediction | 10,262 | 28.7 (27.1-30.3) | 3.77 (3.49-4.07) |

^a^ The incidence rate is expressed in 1,000 person-years.

Abbreviations: CI, confidence interval; DNN, deep neural network; HR, hazard ratio; No., number

**Supplementary Figure 8.** Age- and sex-weighted Kaplan–Meier curves for incident left ventricular systolic dysfunction (LVSD) among patients in subset B stratified by (a) DNN-signal predictions and (b) DNN-image predictions.

| **(a) Using signal** | | | | **(b) Using image** | | | |
| --- | --- | --- | --- | --- | --- | --- | --- |
| 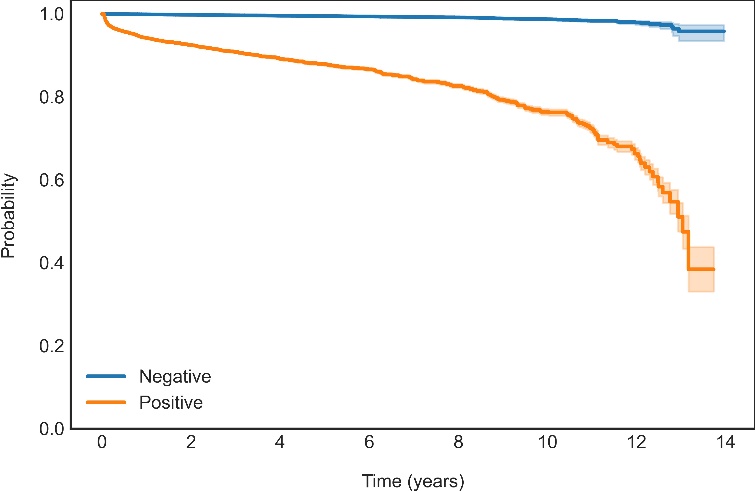 | | | | 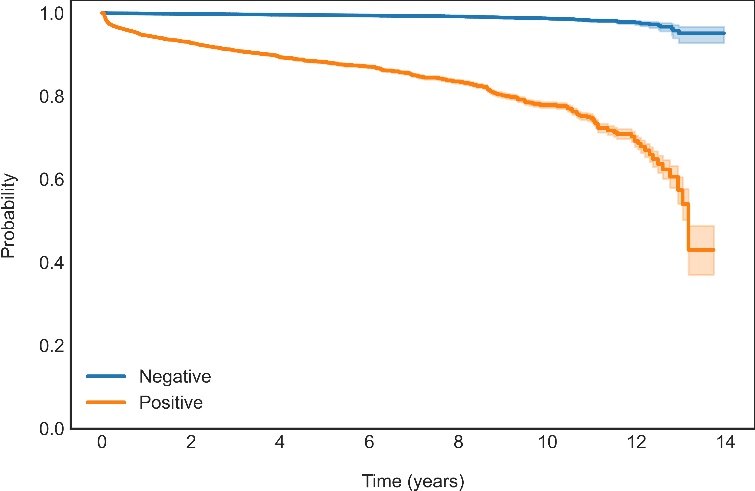 | | | |
|  | **No. of patients** | **Incidence rate** ^a^  **(95% CI)** | **Adjusted HR**  **(95% CI)** |  | **No. of patients** | **Incidence rate**^a^  **(95% CI)** | **Adjusted HR**  **(95% CI)** |
| Negative prediction | 74,928 | 1.2 (1.1-1.4) | 1.00 (Reference) | Negative prediction | 73,795 | 1.2 (1.0-1.3) | 1.00 (Reference) |
| Positive prediction | 8,859 | 38.2 (35.6-40.9) | 26.35 (22.95-30.25) | Positive prediction | 9,992 | 35.5 (33.1-38.0) | 24.83 (21.56-28.59) |

^a^ The incidence rate is expressed in 1,000 person-years.

Abbreviations: CI, confidence interval; DNN, deep neural network; HR, hazard ratio; No., number

**Supplementary Figure 9.** Age- and sex-weighted Kaplan–Meier curves for all-cause mortality among patients in subset B stratified by (a) DNN-signal predictions and (b) DNN-image predictions.

| **(a) Using signal** | | | | **(b) Using image** | | | |
| --- | --- | --- | --- | --- | --- | --- | --- |
| 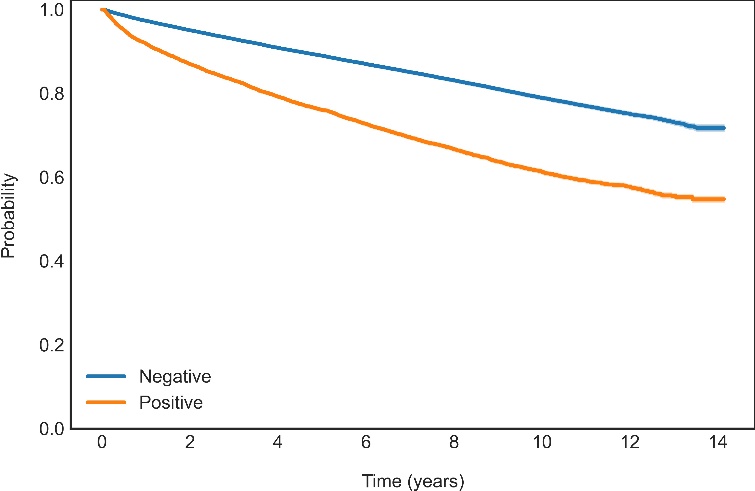 | | | | 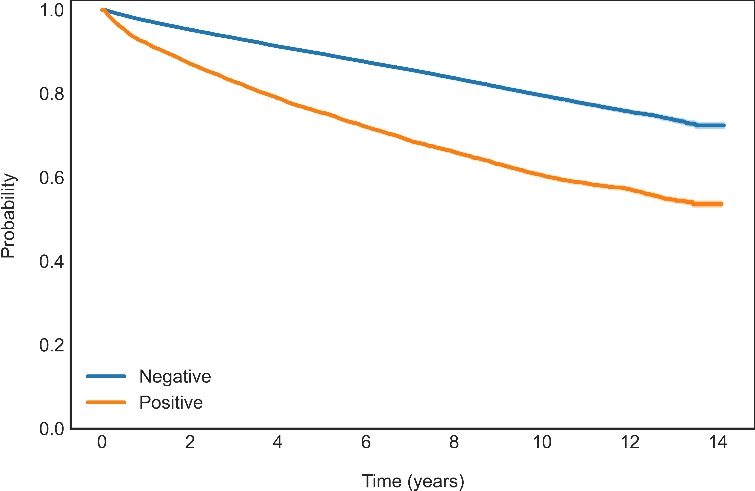 | | | |
|  | **No. of patients** | **Incidence rate** ^a^  **(95% CI)** | **Adjusted HR**  **(95% CI)** |  | **No. of patients** | **Incidence rate** ^a^  **(95% CI)** | **Adjusted HR**  **(95% CI)** |
| Negative prediction | 74,928 | 22.9 (22.5-23.3) | 1.00 (Reference) | Negative prediction | 73,795 | 21.7 (21.3-22.1) | 1.00 (Reference) |
| Positive prediction | 8,859 | 62.6 (60.5-64.8) | 2.18 (2.09-2.26) | Positive prediction | 9,992 | 69.8 (67.7-72.0) | 2.32 (2.24-2.41) |

^a^ The incidence rate is expressed in 1,000 person-years.

Abbreviations: CI, confidence interval; DNN, deep neural network; HR, hazard ratio; No., number

**Supplementary Figure 10.** Age- and sex-weighted Kaplan–Meier curves for cardiovascular mortality among patients in subset B stratified by (a) DNN-signal predictions and (b) DNN-image predictions.

| **(a) Using signal** | | | | | **(b) Using image** | | | | | |
| --- | --- | --- | --- | --- | --- | --- | --- | --- | --- | --- |
| 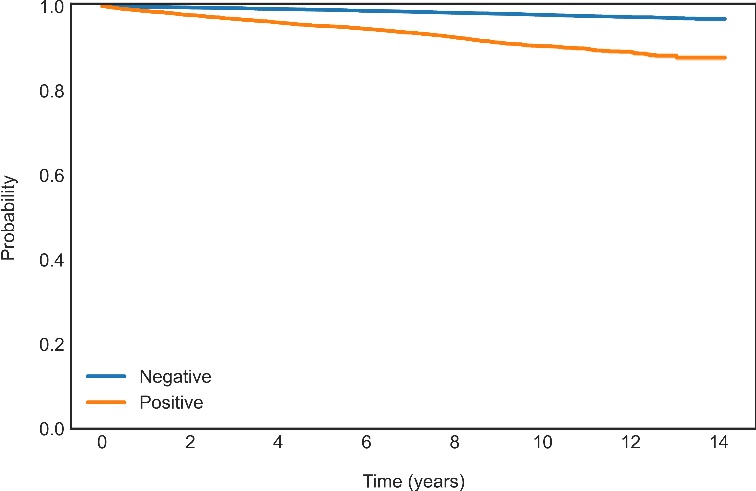 | | | | | 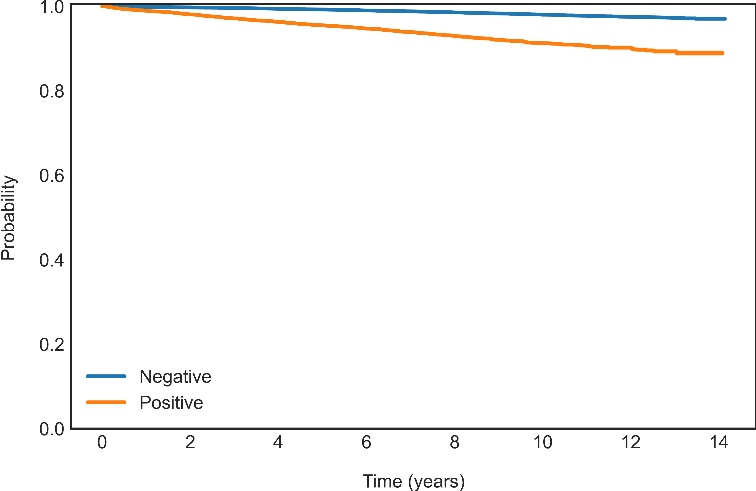 | | | | | |
|  | **No. of patients** | **Incidence rate** ^a^  **(95% CI)** | **Adjusted HR**  **(95% CI)** | |  | | **No. of patients** | | **Incidence rate** ^a^  **(95% CI)** | **Adjusted HR**  **(95% CI)** |
| Negative prediction | 74,928 | 1.9 (1.8-2.0) | | 1.00 (Reference) | Negative prediction | 73,795 | | 1.8 (1.6-1.9) | | 1.00 (Reference) |
| Positive prediction | 8,859 | 12.1 (11.2-13.1) | | 5.20 (4.70-5.75) | Positive prediction | 9,992 | | 12.2 (11.3-13.1) | | 4.99 (4.52-5.52) |

^a^ The incidence rate is expressed in 1,000 person-years.

Abbreviations: CI, confidence interval; DNN, deep neural network; HR, hazard ratio; No., number

**Supplementary Figure 11.** Age- and sex-weighted Kaplan–Meier curves for all-cause mortality among patients in subset C stratified by (a) DNN-signal predictions and (b) DNN-image predictions.

| **(a) Using Signal** | | | | **(b) Using Image** | | | |
| --- | --- | --- | --- | --- | --- | --- | --- |
| 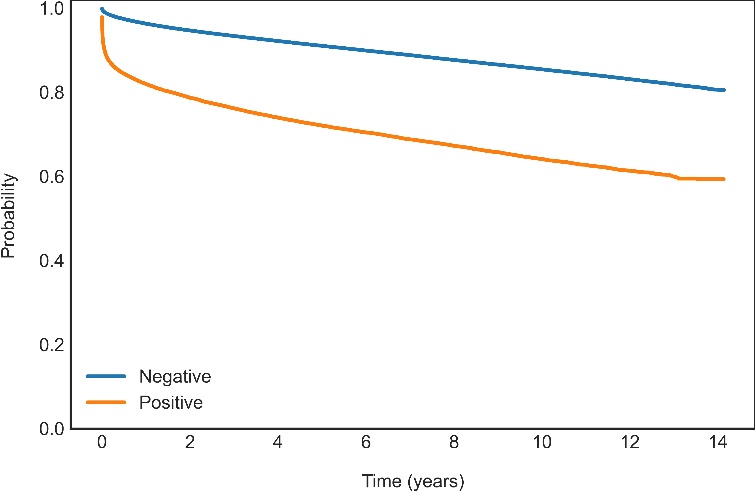 | | | | 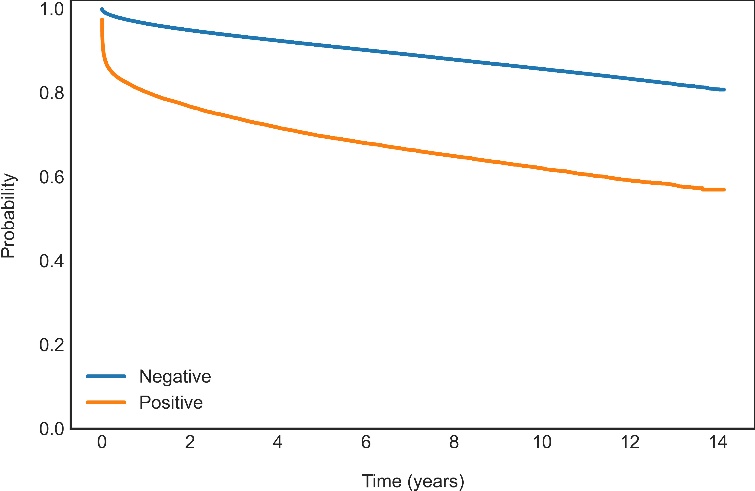 | | | |
|  | **No. of patients** | **Incidence rate**^a^  **(95% CI)** | **Adjusted HR**  **(95% CI)** |  | **No. of patients** | **Incidence rate**^a^  **(95% CI)** | **Adjusted HR**  **(95% CI)** |
| Negative prediction | 1,155,523 | 16.9 (16.8-17.0) | 1.00 (Reference) | Negative prediction | 1,151,691 | 16.3 (16.2-16.4) | 1.00 (Reference) |
| Positive prediction | 39,459 | 100.3 (98.8-101.8) | 3.24 (3.19-3.29) | Positive prediction | 43,291 | 120.5 (118.9-122.2) | 3.46 (3.40-3.51) |

^a^ The incidence rate is expressed in 1,000 person-years.

Abbreviations: CI, confidence interval; DNN, deep neural network; HR, hazard ratio; No., number

**Supplementary Figure 12.** Age- and sex-weighted Kaplan–Meier curves for cardiovascular mortality among patients in subset C stratified by (a) DNN-signal predictions and (b) DNN-image predictions.

| **(a) Using Signal** | | | | **(b) Using Image** | | | |
| --- | --- | --- | --- | --- | --- | --- | --- |
| 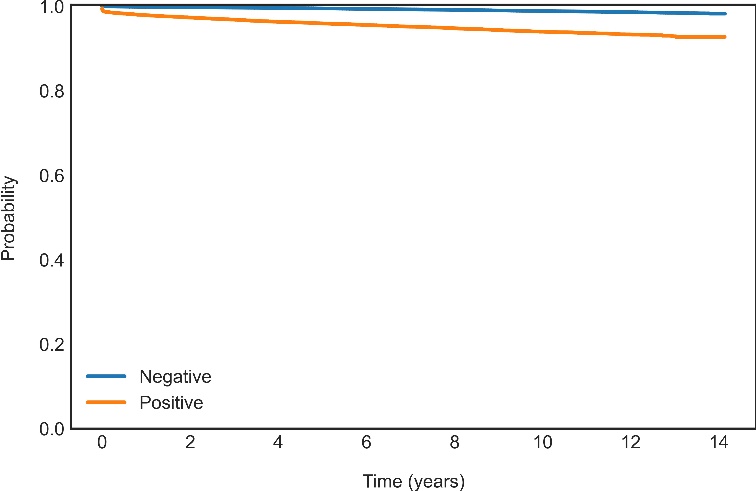 | | | | 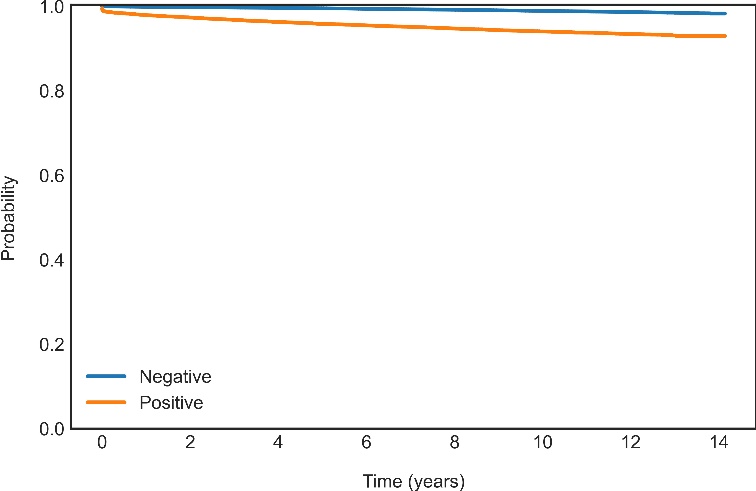 | | | |
|  | **No. of patients** | **Incidence rate**^a^  **(95% CI)** | **Adjusted HR**  **(95% CI)** |  | **No. of patients** | **Incidence rate**^a^  **(95% CI)** | **Adjusted HR**  **(95% CI)** |
| Negative prediction | 1,155,523 | 1.1 (1.0-1.1) | 1.00 (Reference) | Negative prediction | 1,151,691 | 1.0 (1.0 -1.0) | 1.00 (Reference) |
| Positive prediction | 39,459 | 14.3 (13.7-14.9) | 6.83 (6.51-7.16) | Positive prediction | 43,291 | 15.9 (15.4 -16.5) | 6.82 (6.51-7.14) |

^a^ The incidence rate is expressed in 1,000 person-years.

Abbreviations: CI, confidence interval; DNN, deep neural network; HR, hazard ratio; No., number

**Supplementary Table 1.** Characteristics of patients in Tri-Services General Hospital

| Characteristics | Training  (n = 91,425) |
| --- | --- |
| Age years, mean ± SD | 62.3 ± 17.4 |
| Age groups, n (%) |  |
| <40 | 10,945 (12.0) |
| 40-49 | 8,433 (9.2) |
| 50-59 | 14,781 (16.2) |
| 60-69 | 20,255 (22.2) |
| 70-79 | 15,080 (16.5) |
| 80+ | 21,931 (24.0) |
| Sex, n (%) |  |
| Female | 45,245 (49.5) |
| Male | 46,180 (50.5) |
| EF, mean ± SD | 65.8 ± 10.5 |
| EF<40%, n (%) | 2,812 (3.1) |
| In-hospital mortality, n (%) |  |
| Within 1 year | 3,083 (3.4) |
| Within 3 year | 4,545 (5.0) |
| Within 5 year | 5,188 (5.7) |
| Anytime | 5,641 (6.2) |

**Supplementary Table 2.** AI-predicted models from the CGMH and the Tri-service General Hospital.

| **Database** | **Sensitivity** | **Specificity** | **False positive**  **rate** | **False negative**  **rate** | **Positive predictive value** | **Negative predictive value** | **F1-score** | **Accuracy** | **Threshold** | **AUC** |
| --- | --- | --- | --- | --- | --- | --- | --- | --- | --- | --- |
| CGMH | 0.91 | 0.86 | 0.14 | 0.10 | 0.22 | 0.99 | 0.36 | 0.86 | 0.43 | 0.95 |
| Tri-service hospital | 0.90 | 0.87 | 0.13 | 0.10 | 0.19 | 0.99 | 0.30 | 0.87 | 0.32 | 0.95 |

Abbreviations: AUC, Area under the receiver operating characteristics curve
